# Beyond Local Domains: Connective Ontology in (Post-)Cognitive Sociology

**DOI:** 10.1177/13607804241237768

**Published:** 2024-06-10

**Authors:** James Rupert Fletcher

**Affiliations:** The University of Manchester, UK

**Keywords:** affinities, cognitivism, domain-specificity, enactivism, localisation

## Abstract

Ontology in cognitive science has long been dominated by cognitivism, developing computer science metaphors to position cognition as intrinsic mind-brain information-processing. Contemporary cognitivism hypothesises localised domain-specificity, disaggregating cognition into discrete subtypes, each of which exists in a dedicated brain region. Latterly, peripheral cognitive science scholarships have contested these ideas, cultivating post-cognitivist dispositions with radical ontologies, relocating cognition in active socio-material ecologies. Nonetheless, much cognitive sociology retains cognitivist ontology, treating sociological phenomena as extrinsic constraints that influence the mind-brain’s foundational cognition. I argue that cognitive sociology could fruitfully engage with post-cognitivist science. As an example, I use connective ontology, from the sociology of personal life, to conceptualise cognition as dynamically emergent and vitally animated ecological connective energies. Doing so, I show that post-cognitivism offers routes towards genuine social ontologies of cognition as a sociological matter, moving beyond cognitivism.

## Cognitive(-ish) sociology

Over three decades, cognitive sociology has emerged as a distinct field, yet has remained tangential to the dominant, and far more cross-fertilising, cognitive science disciplines of cognitive neuroscience and cognitive psychology. Today, cognitive sociology contains several sub-traditions inspired by well-known sociological traditions, for example, Bourdieu, Goffman, Durkheim (Brekhus, 2015). Intersecting most cognitive sociology are commitments owing to Berger and Luckmann’s (1966) work on the social construction of knowledge, which underpins the ontological relationship between cognitive sociology, the other cognitive sciences, and cognitivism. Much cognitive sociology accepts, implicitly or explicitly, a cognitivist ontology wherein cognition is foundationally mind-brain-based information-processing, and pursues questions relating to the subsequent social determination of cognition by extrinsic factors, or unidirectional aggregation from foundational mind-brain cognition to downstream social phenomena. Such work effectively approaches cognition as something beyond sociology, ontologically belonging to the neuroscience-psychology nexus, and subsequently shaping and shaped by social forces, which are the proper domain of sociology. Regretting this tangentiality of cognitive sociology to cognitive science, some argue that sociology should double-down on cognitivism to become a more legitimate player in mainstream cognitive science (Lizardo, 2014; Vaisey, 2021). These arguments: first, reiterate long-standing critiques of sociology as ideologically constructionist, relativistic, pseudo-scientific, and anti-naturalistic, and second, suggest that alignment with cognitive science would provide political, institutional, and financial security for sociology.

Such arguments overstate how dogmatic and endangered sociology is, discounting exciting developments across feminism, post-humanism, new materialism, the ontological turn and biosocial sociology that prioritise matter and natural processes, without essentialising away the social (Pitts-Taylor, 2014). For example, Latour’s (2005) actor-network theory (ANT) centres real-world dynamic relations between things and ideas, rather than assuming overarching social forces, while Braidotti’s (2013) post-humanism replaces the idea of the self-contained cognitive individual with multiple identities emerging in relation to the wider material and ideational world. These scholarships – rooted in contemporary sociologies that do not take cognitivism for granted – might offer alternatives to cognitive sociology’s relationship with cognition as a research problem. Furthermore, cognitive sociology’s implicit cognitivism, and arguments for making it more explicit, similarly discounts contemporary post-cognitivist cognitive science, which I will outline shortly.

My point here is that several intellectual scaffoldings already exist that could facilitate reconfigurations of cognitive sociology and its relationship with cognition as a research problem. I argue that one way in which cognitive sociology could pursue different social ontologies of cognition is by tapping into the marked resonances between monist ontological work in sociology and post-cognitivist work in cognitive science. To exemplify my contention, I develop Mason’s (2011, 2018) connective ontology, a monist ontology taken from the sociology of personal life, as an example of a sociological ontology of cognition that echoes contemporary post-cognitivist scholarships. Monism is helpful in this instance because it resists hierarchal ontologies whereby sociological phenomena are subsequent and additional to foundational neural-psychic cognition.

Importantly, I am not suggesting that constructionist or dualist commitments are altogether inconducive to worthwhile cognitive sociology. These also come in varied gradations, for example, Fairclough et al.’s (2013) arguments for realist sociologies that take semiotics seriously through a ‘weak’ constructionism, which principally differ from the material turn by demarcating semiotics as a distinct category. Here, my argument is simply that resonances between emerging post-cognitivist cognitive science and contemporary monist social thought could provoke new cognitive sociologies that need not rely on cognitivist ontology, hopefully begetting a richer plurality of approaches. That plurality would, nonetheless, be further enriched by other perspectives.

In this article, I first explicate cognitivist ontology in cognitive science, focussing on domain-specificity and localisation as exemplifying cognitivism’s taxonomic impulses. Second, I outline post-cognitivist responses to that cognitive science, extending cognition beyond individual mind-brains and into living ecologies. Finally, I show that connective ontology mirrors post-cognitivist sensibilities in an established sociological form and can thereby render cognition itself an essentially sociological entity. This argument repositions sociology as a core and distinct discipline of cognitive science, soothing fears of institutional irrelevance without abandoning sociological sensibilities.

## Cognitivist domain localisation

As computer science developed in the 20th century, stakeholders likened computers to synthetic brains (Copeland, 2012). Early cognitive scientists mirrored these popular metaphors, establishing the mind-brain as an empirical problem by likening it to information-processing software running on neurological hardware (Abraham, 2016). This metaphorical cross-fertilisation came to characterise cognitivism, proposing that cognition is information-processing in the brain (Fletcher, 2023a, 2023b). Contemporary cognitivism rests on interrelated beliefs in domain-specificity (i.e. cognition is made up of subtypes, for example, motor skills, language) and localisation (i.e. those subtypes happen in dedicated bits of brain) (Anderson 2014).

Appeals to domains (e.g. memory, attention, perception) are commonplace within and beyond cognitive science (Harvey, 2022). Domain-specificity echoes long-standing philosophical commitments to mental taxonomy, from Aristotle to Kant, wherein thought is described as existing in discrete types (Hacking, 2001). This philosophy underpinned 18th-century phrenology, for example, Thomas Reid typified *lust*, *resentment*, and *musical ear* (Nichols, 2007), Franz Josef Gall offered *smell*, *memory*, and *benevolence* (Anderson, 2014), George Combe described *combativeness*, *secretiveness*, and *veneration* (Van Wyhe, 2004). Phrenology waned in the 19th century, but domain taxonomies re-emerged through 20th-century cognitivism. Chomsky (1980) attributed distinct types of language to types of cognition and Fodor (1983) generalised across cognition per se. The basic concept of cognitive domains transitioned from an epistemological tool for analysing cognition into an ontological claim about what cognition was, eventually dominating cognitive science (Pietraszewski and Wertz, 2022).

Localisation calcifies domain-specificity by positing that domains exist in corresponding bits of the brain. The clear affinities between domain-specificity and localisation, aligning with computational metaphors, have become co-intensifying in cognitivist cognitive science (Hacking, 2001). Like domain-specificity, localisation rearticulates 18th-century phrenology, for example, Combe’s mental faculties occupied dedicated ‘organs’ of the brain (Anderson, 2014). Localisation also enjoys a history of brain injury evidence, whereby neuro-morphological insults correlate with psycho-behavioural change. While infamous accounts have been critiqued for relying on artful interpretations, they remain potent exemplars of domain localisation in cognitive science, substantiating cognitivism (Kotowicz, 2007; Macmillan and Lena, 2010).

Today, cognitivism informs circular cognitive science experimentation predicated on domain localisation: when we are interested in a brain region, we ask what processes it is responsible for; when we are interested in a cognitive function, we ask where in the brain it happens (Fletcher and Birk, 2019). Any domain or location can provide a reference point for assessing its respective neural or psychic analogue (Poldrack and Yarkoni, 2016). In the 1980s, Chomsky’s cognitive modules were justified vis-à-vis localisation (Hacking, 2001). If phrenologists had had the technologies, we might have had in-vivo blood flow evidence for *lust*, *resentment*, or *musical ear*. Beyond this circular logic, the existential nature of domain-localisation – which casts cognitive functions as the purposes of their respective brain bits – is similarly suspect. This metaphysics permeates the computational metaphors that characterise cognitivism, because computers are designed by intelligent creators, with components predestined for certain tasks. Echoes of purpose-driven design in cognitivism endow cognitive science with a pseudo-creationism, wherein brain regions have purposes, contradicting evolutionary conceptions of ecology-responsive adaptation. Hence, arguments that cognitive sociology could become more scientistic by embracing cognitivism are questionable (Anderson, 2014).

## Evolution, ecology, extension

Theoretically, its characteristic pseudo-creationism troubles advocations of cognitivism as a fix for anti-naturalism. More empirically, domain localisation is also in tension with evidence. Locations and domains have been demonstrably associated in multiple variations, for example, the insula has been linked to pain, memory and disgust, among other things (McCaffrey, 2015), and the once facial-recognition-dedicated Fusiform Face Area has now been associated with various non-facial stimuli (Anderson, 2014). Domain localisation has also been experimentally discredited in sum. A study of 11 domains and 78 locations found that each location was typically implicated in nine domains (Anderson and Pessoa, 2011). Moreover, such evidence is itself biased towards domain localisation because it assumes the existence of domains and locations to test relationships, discrediting domain localisation by showing alternative domain-location relations.

The neural reuse hypothesis responds to this evidence, suggesting that cognition is supported across perpetually transforming neural networks adapting to circumstantial requirements. Here, there is some basic location and domain specificity, and bias towards instances of domain localisation, but far greater context-dependent reconfiguration and fluidity (Anderson, 2014). By analogy, I mostly connect my house and my office by cycling, but occasionally I walk if I have plans (or a flat tyre). When my parents visited and it rained, we attempted to catch the bus, but it never arrived, so we caught a taxi instead. One time, a friend drove me because I had bulky items to collect. Ultimately, my home-to-office connections are mostly realised through bike, but various alternatives are possible in response to situational peculiarities.

Bridging neural reuse and evolutionary theory, several evolutionary psychologists have foregrounded plasticity over domain localisation, whereby the mind-brain is adaptable to circumstance, as the primary form of evolved neuropsychological fitness (Pietraszewski and Wertz, 2022). Herein, the mind-brain is brought into direct relation with the world vis-à-vis evolutionary principles, while simultaneously escaping pop-psych notions of now-inherent cognitive characteristics having evolved in response to historic circumstances (Barrett, 2015). This ecological responsiveness offers a more naturalistic representation of cognition. However, it remains centred on the mind-brain and somewhat alienated from traditional sociological terrains.

Potentially offering solutions to this problem, new post-cognitivist ontologies of cognition are echoing naturalistic commitments to ecological responsiveness in radical ways (e.g. De Haan, 2020; Lobo, 2019; Newen et al., 2018). These scholarships are pushing cognition beyond mind-brains, and beyond individuals, into ecologies. Anderson (2014) exemplifies this post-cognitivism through Scrabble, wherein players manoeuvre letters in their stand to reveal the best words, combining their fingers, the letters, and the stand to do word selection. The staunchest post-cognitivist tradition is radical enactivism, which dismisses representationalism and conceptualises cognition as a natural ecological process. Radical enactivism is commonly exemplified by a slinky, which can deftly negotiate stairs because of the relations between its own and its environment’s material characteristics (Hutto and Myin, 2017). By analogy, ecological cognition emanates from natural relations between a range of material entities – body parts, technologies, places, and so on.

While newly radicalised and articulated as such, these sorts of holistic critiques of mainstream cognitivism in cognitive science can be charted back to the 1990s in the work of Clark and Chalmers (1998), who first advocated the idea that cognition extended beyond the human subject. Concurrently, Edwards’ (1997) discursive approach critiqued mainstream psychology for misleading cognitivist typologies, failing to attend to real-world manifestations of cognition as non-categorial and dynamically enacted in multiple forms. Hence, cognitivism’s discontents stretch back several decades.

Today, ecological cognition has been empirically demonstrated in several forms, for example, ‘embodied’, ‘enacted’, ‘extended’. Evidencing embodiment, successful traders are more reliant on interoception (perception of internal bodily sensation) than intelligence (Coates, 2012). Evidencing enaction, radiologists review scans more quickly and accurately when moving compared with being still (Fidler et al., 2008). Evidencing extension, people with cognitive impairments can maintain high-level function by outsourcing cognition to digital technologies (Annese et al., 2022). Together, such instances realise a version of ecological responsiveness by positioning cognition as something that exists in ecologies beyond the mind-brain.

Post-cognitivist appeals to ecology as an alternative to cognitivism can satisfy both naturalistic and sociological predilections, undermining aforementioned critiques of irreconcilability. However, a word of caution here. Pockets of social science have, in recent years, eagerly subscribed to scientific theories that initially appeared to substantiate sociological convictions, for example, mirror neurons (Mihara, 2010), epigenetics (Landecker and Panofsky, 2013), and microbiomes (Ignatow, 2021). Such ideas have typically floundered, suggesting a tendency to too readily accept extra-disciplinary hypotheses that feel conducive to sociological projects. I am therefore cautious about simply adopting radical enactivism, or something similar, outright. Instead, cognitive sociology might fruitfully develop its own post-cognitivist ontologies of cognition. I suggest that *connective ontology*, taken from the sociology of personal life, can furnish an ontologically engaged cognitive sociology.

## Connective cognition

Connective ontology sits within a broad ontological turn in sociology over recent decades, rejecting traditional disciplinary dualities and debates between constructionism and materialism (Pickering, 2017). Instead, the ontological turn has offered flat ontologies foregrounding relationalities, blending inspiration from quantum physics and indigenous metaphysics (Gullion, 2018). Herein, sociologies have made ideas such as entanglement, assemblage, and intra-action central to their ontological schemas, articulating a fluid world made up of relationships (Fox and Alldred, 2016; Giraud, 2019). Like post-cognitivism, cognitive sociology has been isolated from the ontological turn. This is regrettable because the ontological turn and post-cognitivism seem ripe with affinities, making human life relational, active, and ecological.

Connective ontology originates in a 2011 paper by Jennifer Mason advocating facet methodology, describing a complex, relational and vital world made up of connection. Facet methodology has subsequently spread across sociology (e.g. Barnwell, 2022; Fletcher and Birk, 2019; Meckin and Balmer, 2021; Phoenix and Bell, 2019), but the connective ontology underpinning it has been largely ignored, at least explicitly. In response, I (Fletcher, 2023c) have redeveloped connective ontology by reinterpreting Mason’s (2018) subsequent scholarship, which implicitly expands connective ontology, through relevant ontological, vitalist, and biosocial sociologies.

Connective ontology presents a world comprising dynamically emergent vitally animated connections. Here, connections are the fundamental units of social reality and hence sociological study. Connections are flows, charges, forces, and energies, vitalised by, and themselves vitalising, other connections. Connective ontology is anti-reductionist and anti-taxonomic. Connections cannot be limited to, nor defined in terms of, the entities (e.g. objects, ideas, people, places) that they intuitively appear to connect. To isolate connection is to disconnect it and hence abolish it, so successful connective sociology must be holistic. Connections are ecological insomuch as they transcend people, flowing through and beyond the things that are connected.

There is much here that resonates with the critical geographies of thinkers such as Massey (2005), whose relational spatial work demarcates processual material–ideational worlds formulated by shifting relationships. In connective ontology, a more overtly sociological bent suggests that those connections owe much of their vitality to humans living our lives. Central to this pseudo-humanism are sensations, which emanate in ecologies and lend a potency to connections by virtue of their affective potentials. Sensations are not attributable to the things sensed or the things sensing, but are dynamically emergent in our ecological connections. Indeed, they are the experiential mediums of our being *in* and *of* our ecologies.

As noted, cognitive sociology has proved immune to the ontological turn, but I argue that two aspects of connective ontology could puncture this bubble and reposition cognition as a genuine post-cognitivist sociological subject. First, the focus on sensations as dynamically emergent in ecologies resonates with post-cognitivist appeals to ecological cognition, while also bridging traditional cognitive science concerns vis-à-vis the senses. Second, the centrality of iterative fluid connection echoes evolutionary appeals to neural reuse, plasticity, and enactivism as modes of ecological responsivity. These two features of connective ontology render it a potential catalyst for ontologically attentive cognitive sociologies, wherein cognition is a decidedly sociology matter, freed from cognitivism.

Working with connective ontology, we might approach cognition as connection in an ontological sense. Such a recalibration of ontological commitments in cognitive sociology would require a refocusing on the connective energies that make up cognition as it is lived ecologically. Doing so could foster new intellectual connections between post-cognitivism and the ontological turn, underpinning a cognitive sociology with a strong social ontology of cognition. Not all connections are cognition, but connective ontology can be helpful here in drawing our attention to emanating sensations as the core connections of what we approach as cognition. Such sensations partially map onto more traditional notions of cognition, but they are also connective energies that exist as much in ecologies as any mind-brain. There are affinities here with network approaches to higher order cognitive systems, studied in sum because their dynamic complexity is their most important operative characteristic, doing cognitive work that transcends their components (Badcock et al., 2019; Van Der Schyff et al., 2018).

As an example, take my hearing as I type this sentence. I hear each key being pushed down and bouncing back up, the taps amassing into the familiar sound of typing. There are obvious connections here, between the plastic of the keys, the air density, and my own aural disposition. However, we are already localising and domaining this cognition, as ‘hearing’ per se, made up of components parts. Hearing does not really work like this. Even simplistically, we know that our aural experiences are co-dependent on visual and other experience (McGurk and MacDonald, 1976). The tapping that I hear relies on what I am seeing, feeling, and surrounded by to varying extents. This opens up questions of real-world sensation. When I write of ‘hearing keys tapping’, I am artificially specifying what, in practice, is far a more complex soundscape, for example, the desktop’s hum, the distant traffic outside the window, a hoover on the floor below. And still this artificially isolates ‘sound’. I feel the key compressions in my fingers, the breeze on my arm nearest the window, bus rumbles vibrating my gut. We could characterise any cognitive phenomenon with similar richness. To ‘hear’ tapping is an impoverished articulation of real-world cognition.

Practically, such a connective disposition could invigorate research by foregrounding methodologies that are rare in cognitive sociology. For instance, multispecies and/or material ethnographies, while intuitively at odds with cognitivist commitments because they decentre individual thought, could be used to implement post-cognitivist cognitive sociologies by deliberately attending to emanating sensations beyond the singular human subject, nurturing empirical engagements with ecological relations as the basic unit of analysis. For instance, considering the above example of tapping keys, we might be less concerned with me hearing and more concerned with the thick web of connections flowing through and with that hearing (perhaps we could begin with the soundscape as subject rather than me). Ultimately, adopting such de-individualised stances towards cognition as a legitimately sociological matter can help us challenge the essentialisation of human action and experience, with important consequences for social justice – e.g, might we reappraise mental capacity legislation, which discriminates based on cognition, if said cognition is fundamentally ecological (Fletcher, 2023d)?

## Conclusion

I began this article by troubling cognitive sociology’s relationship with cognitive science and cognitivism. At best, such sociology seems ontologically agnostic, leaving neuroscience and psychology to dictate the mind-brain, and then introducing sociological concerns after the fact. At worst, it seems existentially fraught, not doing foundational cognitivism and regretting its inability to do so. Some argue that ‘cognitive social scientists should offer explanations of social phenomena that “go all the way down” to the neuronal level’ (Sarkia et al., 2020: 585). Why so? Could the ontological commitments of cognitive sociology not come from contemporary sociologies rather than cognitivism? Connective ontology offers a (but not the only) means for cognitive sociology to reposition itself in relation to cognition. Importantly, I am not suggesting the outright abandonment of cognitivism. Instead, I want to encourage a richer and more heterogeneous sociology of cognition.
